# Resection of peritoneal metastases causing malignant small bowel obstruction

**DOI:** 10.1186/1477-7819-5-122

**Published:** 2007-10-24

**Authors:** Saleh M Abbas, Arend EH Merrie

**Affiliations:** 1 Colorectal Unit, Auckland City Hospital, Private Bag 92024, Auckland, New Zealand

## Abstract

**Background:**

Resection of peritoneal metastases has been shown to improve survival in patients with abdominal metastatic disease from abdominal or extra abdominal malignancy. This study evaluates the benefit of peritoneal metastatic resection in patients with malignant small bowel obstruction and a past history of treated cancer.

**Patients and methods:**

Patients undergoing laparotomy for resection of peritoneal metastases from recurrence of previous cancer between 1992–2003 were reviewed retrospectively. Data were collected about type of primary cancer, interval to recurrence, extent of the disease and completeness of resection, morbidity and mortality and long-term survival.

**Results:**

Between 1992 and 2003 there were 79 patients (median age 62, range 19–91) who had laparotomy for small bowel obstruction due to recurrent cancer. The primary cancer was colorectal (31), gynaecologic cancer (19), melanoma (16) and others (13). Overall, the rate of complications was 35% and mortality was 10%. Median survival was 5 months; patients with history of colorectal cancer had better survival than other cancer (median survival 7 months vs. 4 months; p = 0.02). Multivariate analysis showed that the extent of recurrent disease was the only factor that affected overall survival.

**Conclusion:**

Laparotomy for small bowel obstruction is a worthwhile option for patients with malignant small bowel obstruction. Although it is associated with significant morbidity and mortality it offers a reasonable survival benefit in particular for patients with completely resectable disease.

## Background

Bowel obstruction in patients with history of cancer is caused by recurrence of cancer in 50% of patients [1,2]. In patients with previously treated colorectal cancer with curative intent 10% develop small bowel obstruction; half of them are due to recurrence [2]. Small bowel obstruction due to recurrent cancer is commonly caused by colorectal cancer, ovarian malignancies, and malignant melanoma [3-5]. Small bowel obstruction due to recurrent cancer tends to happen earlier (within two years of the initial surgery) than obstructions caused by band adhesions, which have a median time of occurrence of five years after the initial surgery [3].

Small bowel obstruction secondary to recurrent cancer carries a grim outlook, however surgical intervention offers better palliation to reduce symptoms and has been shown to reduce obstruction recurrence rates [5]. Although it is associated with high morbidity and mortality rates, surgical intervention is generally recommended as the best palliation in such patients [4].

Long-term survival is variable and contributing factors are poorly identified. We conducted this study to assess factors that affect long-term survival, which may help in prognostication and management of patients with metastatic peritoneal disease.

## Patients and methods

Auckland City Hospital database was searched for patients who had small bowel resection for cancer recurrence after previous cancer surgery during the period 1992–2003. Patients were included regardless of primary cancer origin. Data were collected by reviewing patients' files including patient demographics, type of primary cancer, time from first diagnosis of cancer to small bowel resection, overall morbidity and mortality, and length of survival after resection of small bowel obstruction and peritoneal metastases.

Overall survival was calculated and univariate analysis (using Pearson correlation test) was performed to correlate survival with primary diagnosis, interval to recurrence and extent of peritoneal disease. Long rank test was used to compare survival difference between different groups. Multivariate analysis was performed using the Cox regression hazard model to specify factors that have impact on overall survival. Statistics were performed using SPSS^® ^11.5 for Windows (SPSS Inc., Chicago).

## Results

**Figure 1:**
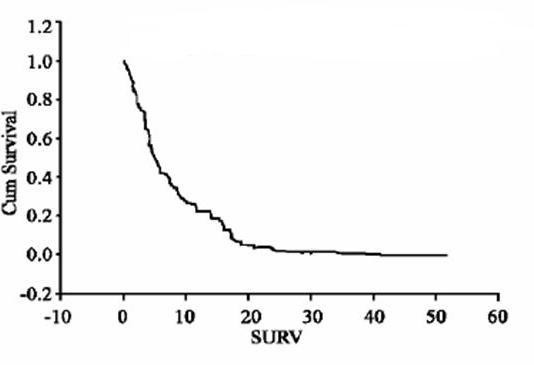
Overall survival for all patients: fraction of surviving patients against months.

Between January 1992 and December 2003, 79 patients (34 males, median age 62, age range 19–91) had laparotomy and resection of peritoneal metastases with or without small bowel resection for peritoneal metastases from intra or extra abdominal cancers (table 1). The most common cause was colorectal cancer followed by gynaecologic cancers. Less common cancers were breast (5), stomach (3), lung (1), prostate (2), and kidney (1). Diffuse non resectable disease was found in 59 patients, 20 patients had limited and resectable disease, which was completely removed. Overall complications rate was 35% and mortality rate was 10%. Median overall survival was 5 months (figure 1).

**Table 1:** Survival by primary malignancy

			Disease extent			
Primary	Number	Median Age	Resectable	Diffuse	Disease free interval (median in years)	Median Survival (months)

Colorectal	31	62	11	20	1.5	7.4
Melanoma	16	51	6	10	5.0	4.4
Gynaecologic	19	58	0	19	1.5	3.7
Miscellaneous	13	67	1	12	1.5	3.5

**Figure 2:**
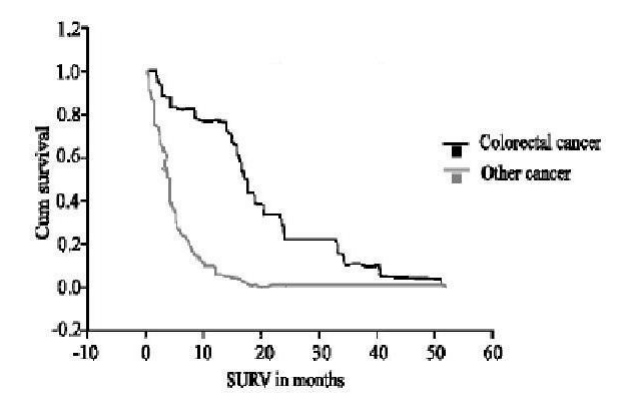
Survival difference between colorectal and other cancers (log rank test). Fraction of patients against months.

Overall survival correlates with type of primary cancer and extent of peritoneal metastases (p = 0.03) (Pearson correlation test). Patients with primary diagnosis of colorectal cancer tend to have less peritoneal disease burden and better overall survival than other types of malignancies (median 7 months vs. 4 months, p = 0.02) (Figure 2).

**Figure 3:**
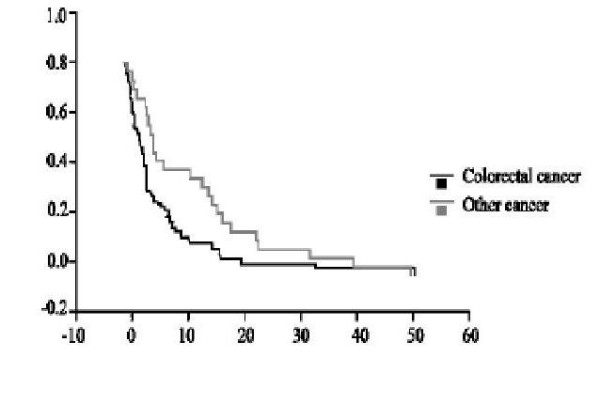
Survival by extent of disease.

Cox regression model of multivariate analysis was used to compare factors that affect overall survival; it showed that among factors that can possibly influence survival, extent of peritoneal disease is the only factor to have an important impact on survival. Median survival for patients with completely resectable disease was 17 months compared with 4 months for unresectable disease (p = 0.002). Age, primary diagnosis and time interval between primary resection and recurrence showed no effect on survival (figure 3).

## Discussion

This study is a retrospective review of 12 years experience with malignant small bowel obstruction. We found that the commonest cause of recurrent malignant small bowel obstruction is colorectal cancer, followed by gynaecologic cancers and malignant melanoma. Malignant melanoma was the commonest extra abdominal malignancy resulting in small bowel obstruction; compared to other cancers it has the longest interval between primary treatment and recurrence (median 5 years). Surgery offered a median survival of 7 months with the best results seen in patients with a primary diagnosis of colorectal cancer, and those with isolated intra abdominal metastases where complete macroscopic clearance of the disease was possible. Multivariate analysis showed that the extent of recurrent disease was the only factor that affected overall survival. Although patients with colorectal cancer had better overall survival this may reflect the fact that it is more likely than other cancers to have completely resectable disease.

Operative mortality has been reported as significantly high particularly in patients who present with cancer recurrence causing acute small bowel obstruction, Walsh et al reported 19% mortality rate [2], other studies reported 24% 30-day mortality rates [3]. Very high death rates of 67% were reported after emergency laparotomy. Surgical intervention in emergency patients is associated with a significant rate of complications; wound complications, intra abdominal sepsis and cardiopulmonary complications are the most common postoperative complications [3]. The reported overall complication rate varies from 8% in patients with melanoma [1] to 44% in gynaecologic malignancies [6]. Median overall survival varies from 5–11 months [2,7,8]. The longest survival is reported in patients with metastatic malignant melanoma by Agrawal *et al*., [1] who described median survival of 15 months after complete resection with five year survival rate of 38%. Malignant melanoma was associated with the longest interval between the primary diagnosis and appearance of peritoneal metastases. Patients with a primary diagnosis of colorectal cancer seem to have better outcome than other cases, with overall survival up to two years [7], however most patients have disease recurrence with two years of resection of the primary colorectal cancer [6].

Currently there is no universally accepted method of follow up abdominal imaging to detect recurrence in those who had colorectal or ovarian cancer resection. Tumour markers such as CEA for colorectal cancer and CA 125 for ovarian cancer are useful tools, however that does not accurately define the time of recurrence, nor quantify the extent of peritoneal disease. CT scan and contrast enhanced CT colonography are useful tools for detection of recurrent colorectal and ovarian and other cancers [5,9,10]. PET is a promising tool for early detection of recurrent intra abdominal cancer when tumour markers are elevated in a symptomatic patient with no signs of recurrence on CT imaging [9,11-14], however this modality is not yet widely available. Future availability of PET may increase the rate of early detection of recurrent disease at stages when it is resectable.

## Conclusion

Metastatic peritoneal recurrence should be suspected in patients with a history of malignancy who present with small bowel obstruction. Aggressive surgical management is recommended in those who prove to have resectable disease, however consideration of this intervention has to be mindful of the high rates of morbidity and mortality. Every effort should be made to pursue a conservative management in those with non resectable recurrence. Future efforts should be directed towards development of strategies for early detection of intra abdominal recurrence.

## Competing interests

The author(s) declare that they have no competing interests.

## Authors' contributions

**SA **and **AEM**: Conceived and designed the study prepared the draft and revised the manuscript for its intellectual content.

All authors read and approved the final manuscript.
